# Supplementary material for: Non-coding somatic mutations converge on the PAX8 pathway in ovarian cancer
Source: Nat Commun. 2020 Apr 24;11:2020. doi: 10.1038/s41467-020-15951-0 (PMC7181647; doi:10.1038/s41467-020-15951-0)
Supplement: Supplementary file 1 — Supplementary Information [file 41467_2020_15951_MOESM1_ESM.pdf]

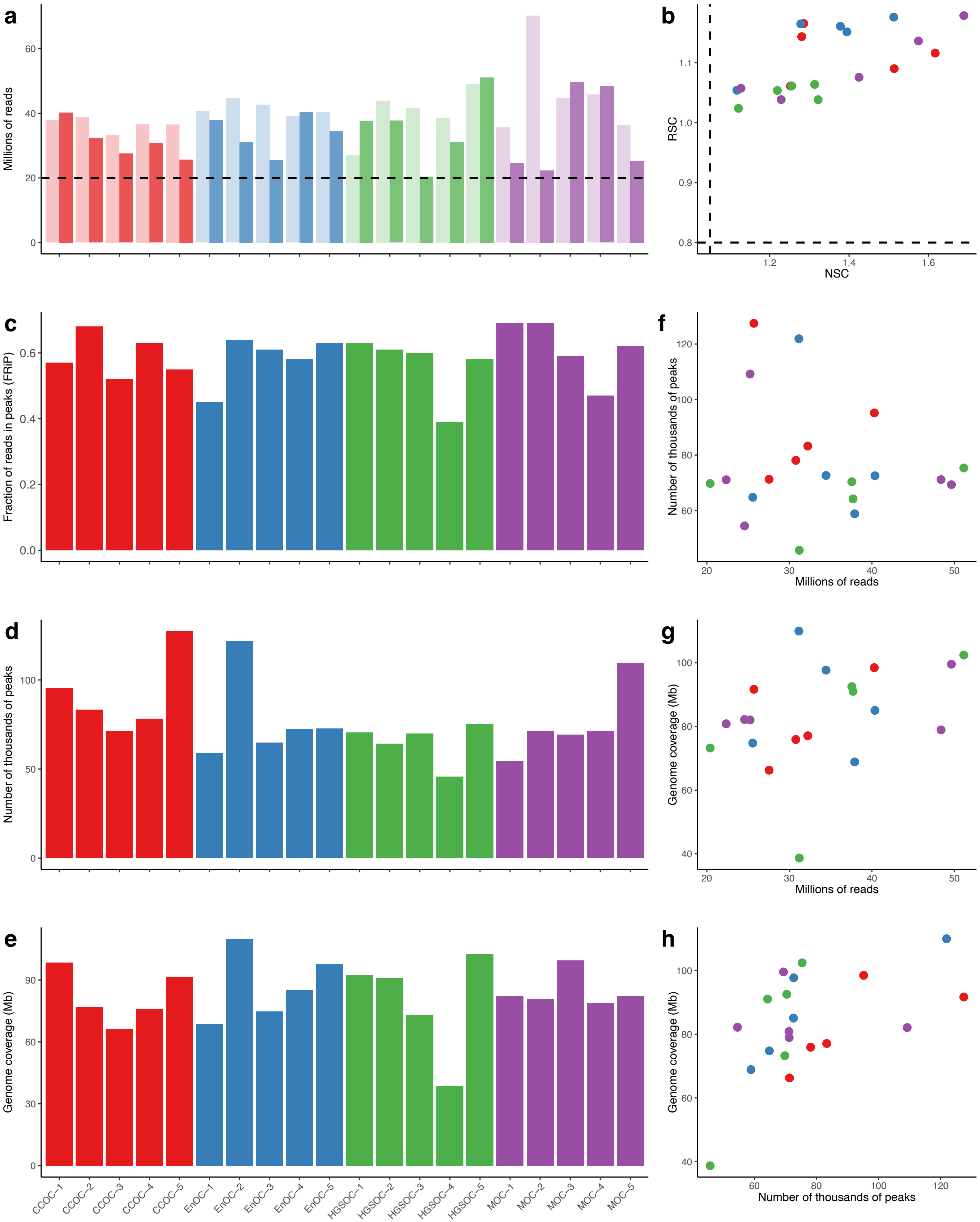


**Supplementary Figure 1.** Quality control metrics for 20 OC H3K27ac ChIP-seq profiles: (**a**) number of reads, (**b**) normalized strand cross-correlation coefficient (NSC) and relative strand cross-correlation coefficient (RSC), (**c**) fraction of reads in peaks (FRiP); and summary of peak calling results: (**d**) number of peaks, (**e**) genome coverage, (**f**) number of reads vs. number of peaks, (**g**) number of reads vs. genome coverage and (**h**) number of peaks vs. genome coverage.

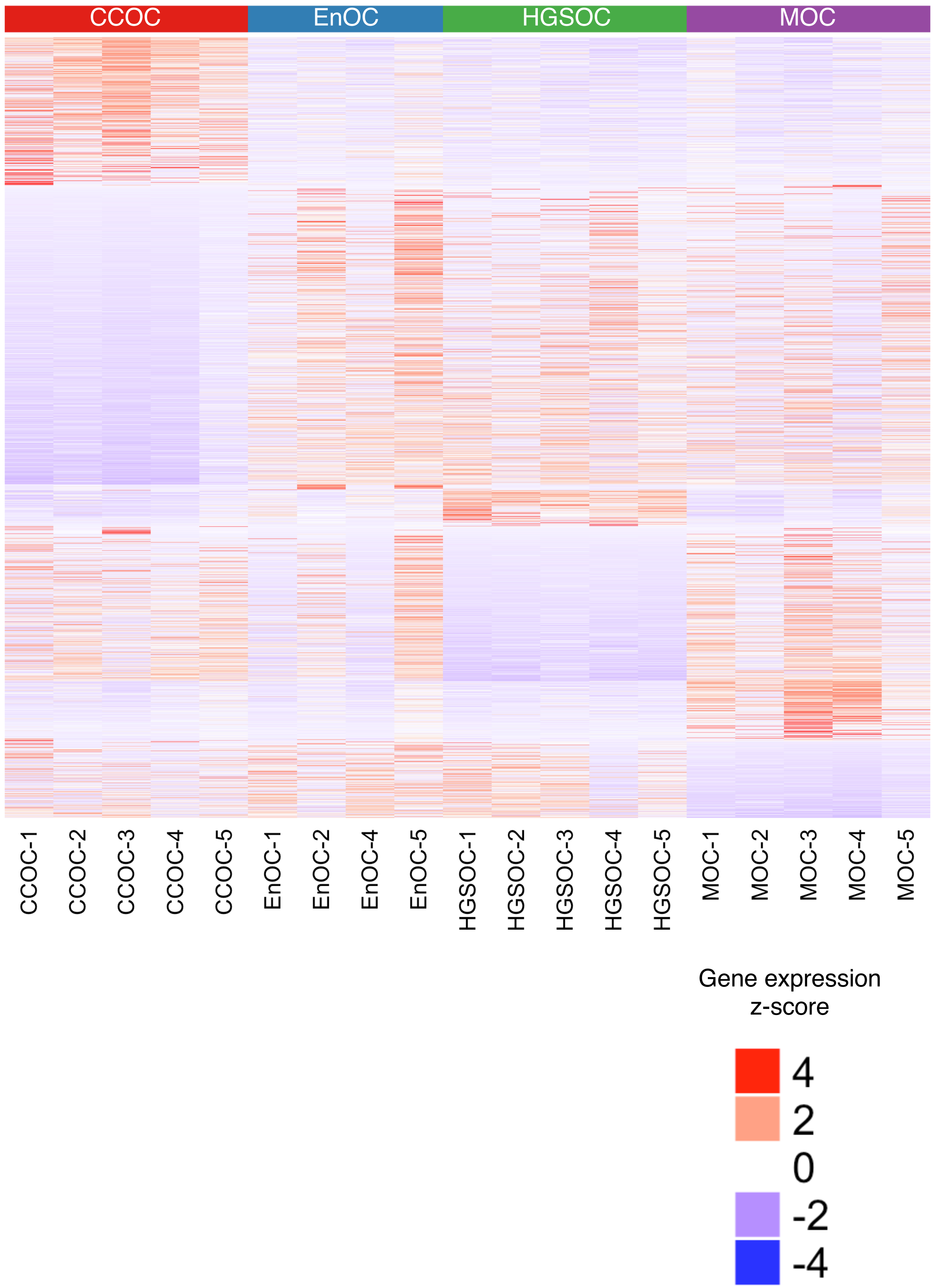


**Supplementary Figure 2.** Histotype-specific gene expression in 19 epithelial ovarian cancers (EOC) from 4 major EOC histological subtypes.

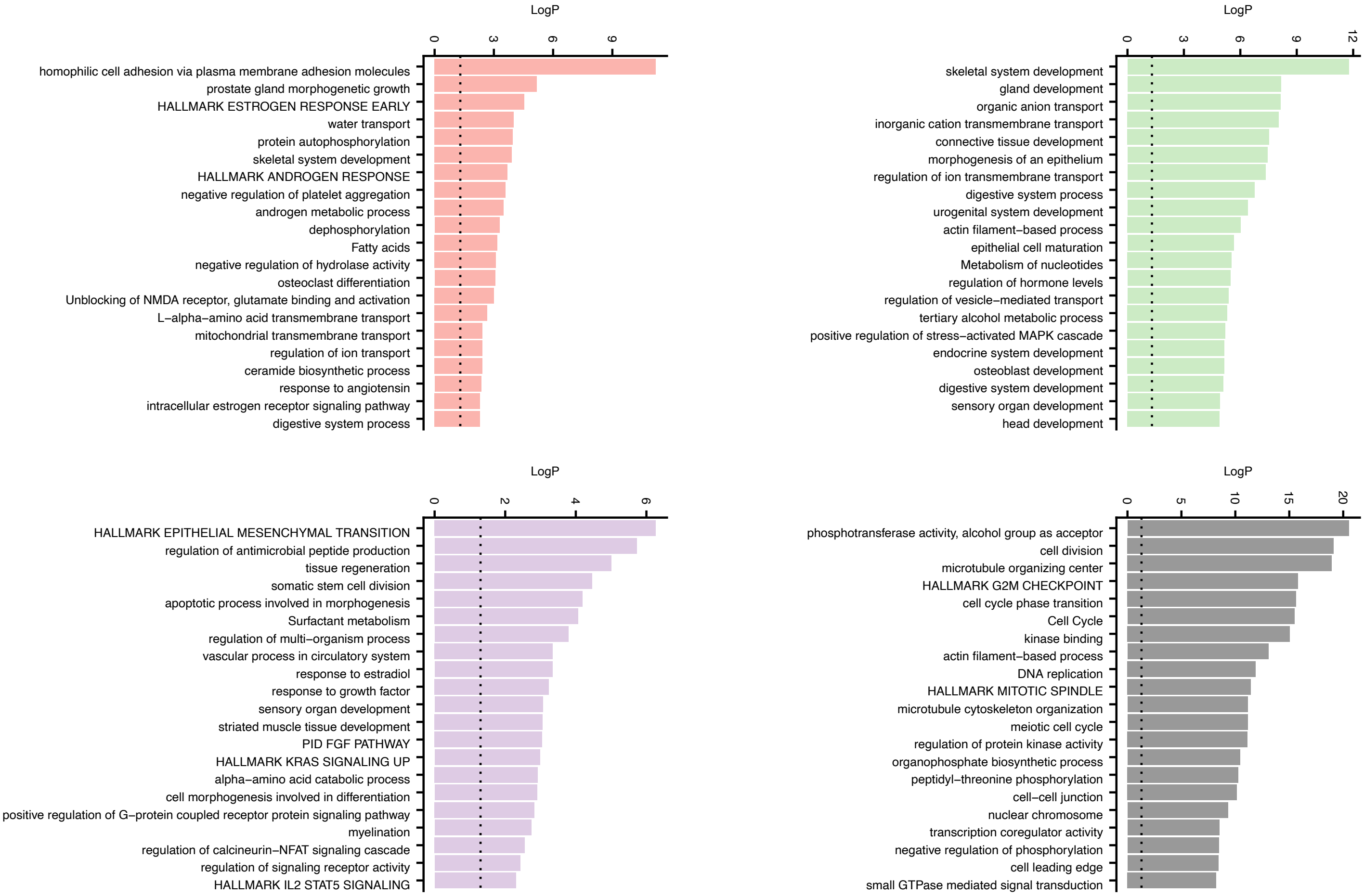


**Supplementary Figure 3.** Pathways enriched in each gene set associated with histotype-specific (Fold change < 0; CCOC – red, HGSOC – green, MOC – purple) and common regulatory elements (REs; gray) epithelial ovarian cancer (EOC).

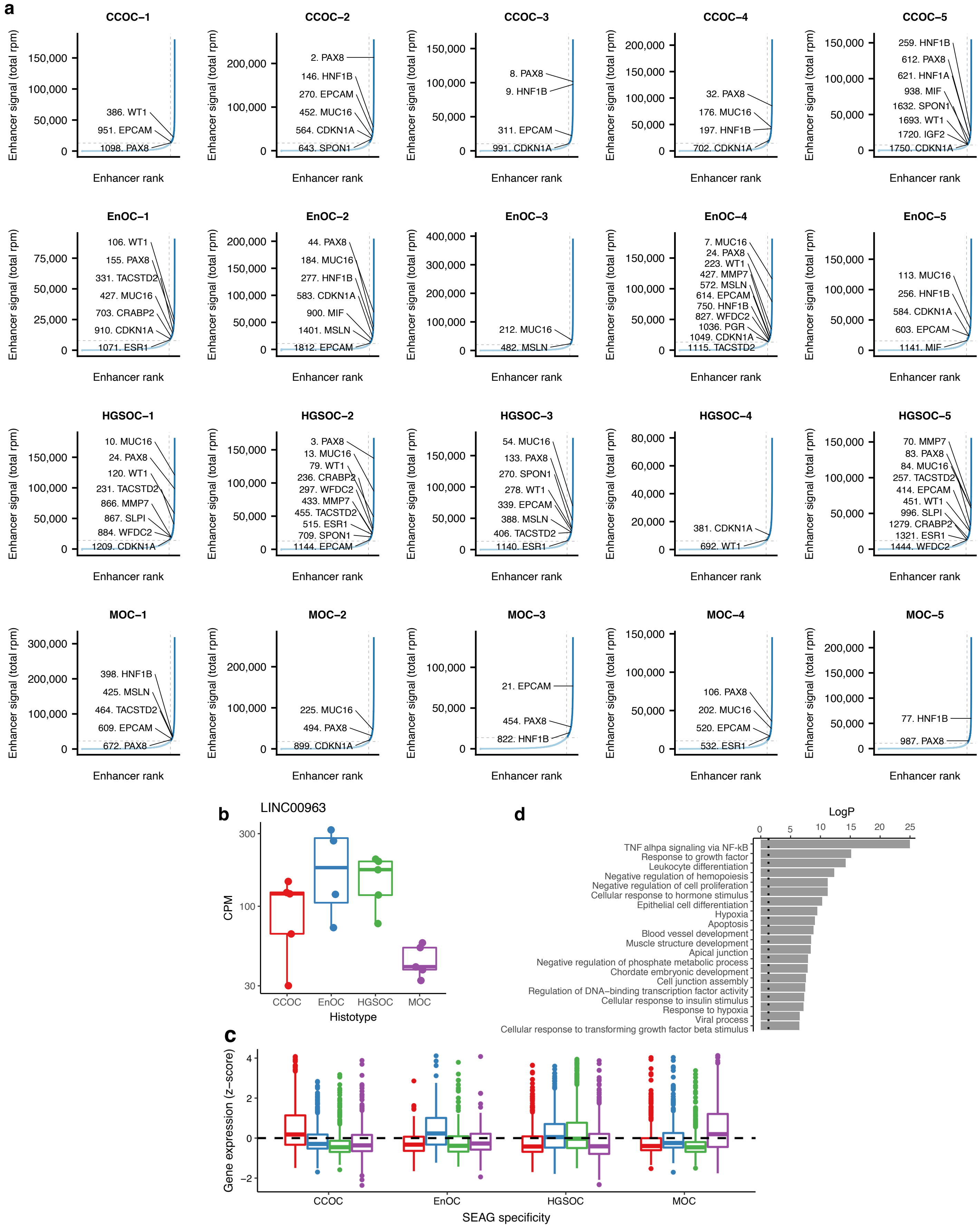


**Supplementary Figure 4.** (**a**) Super enhancer calls in the 20 EOCs. (**b**) *LINC00963* expression in 19 EOCs. (**c**) Normalized gene expression of histotype-specific super-enhancer associated genes (SEAGs). (**d**) Pathway enrichment analysis of SEAGs in EOC.

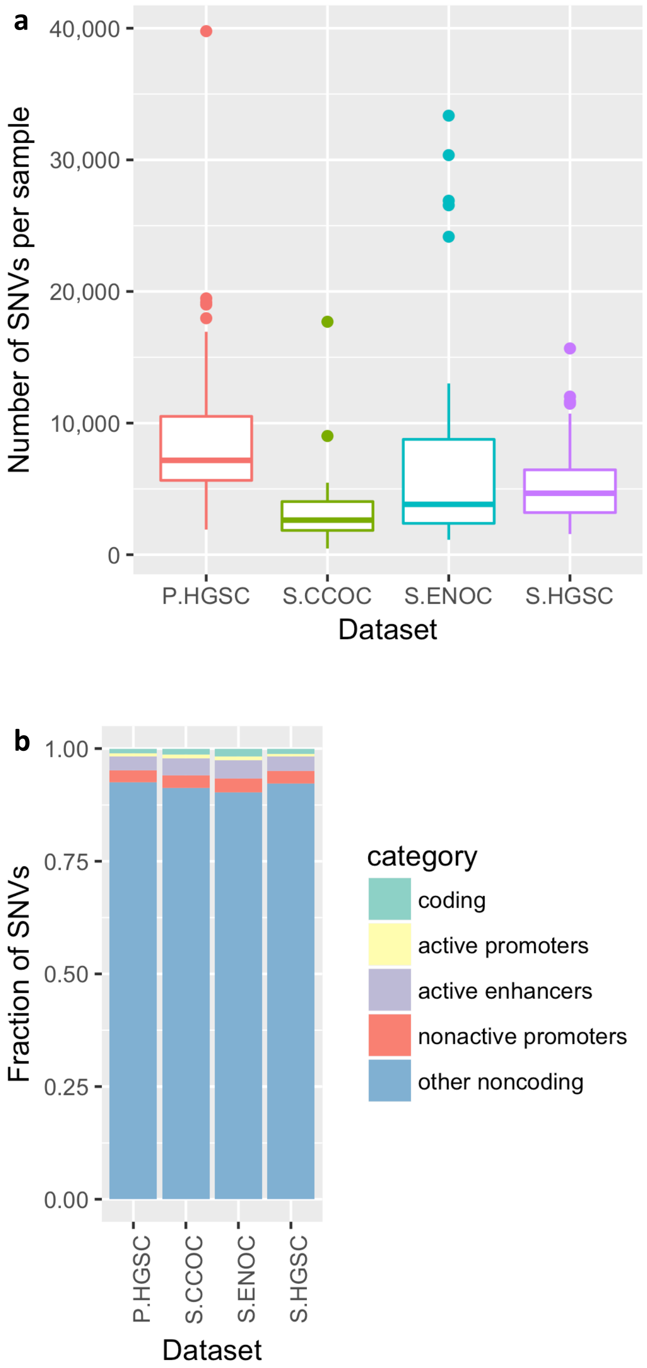


**Supplementary Figure 5.** (**a**) Number of single nucleotide variants per sample and (**b**) distribution of single nucleotide variants by genomic regions: coding, active promoters, active enhancers, nonactive promoters and other noncoding.

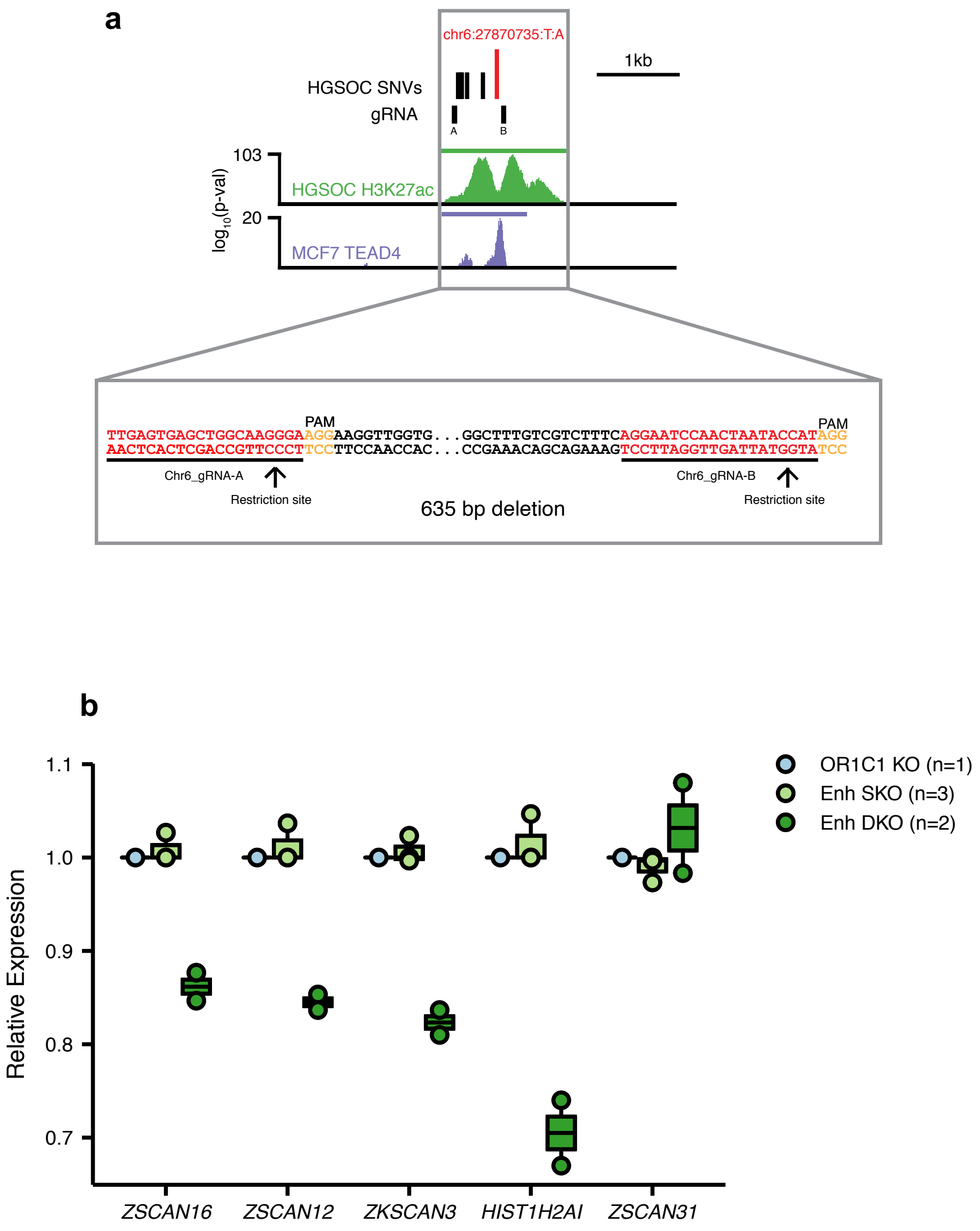


**Supplementary Figure 6.** CRISPR/Cas9 mediated deletion of a frequently mutated regulatory element in HGSOC. (**a**) Diagram showing the gRNA sequence, the PAM and restriction sites. (**b**) Relative expression of *ZSCAN16*, *ZSCAN12*, *ZKSCAN3*, *HIST1H2AI* and *ZSCAN31* by qPCR in single-cell derived clones before and after chromosome 6 enhancer KO in UWB1.289 cell line.

**a**

**b**

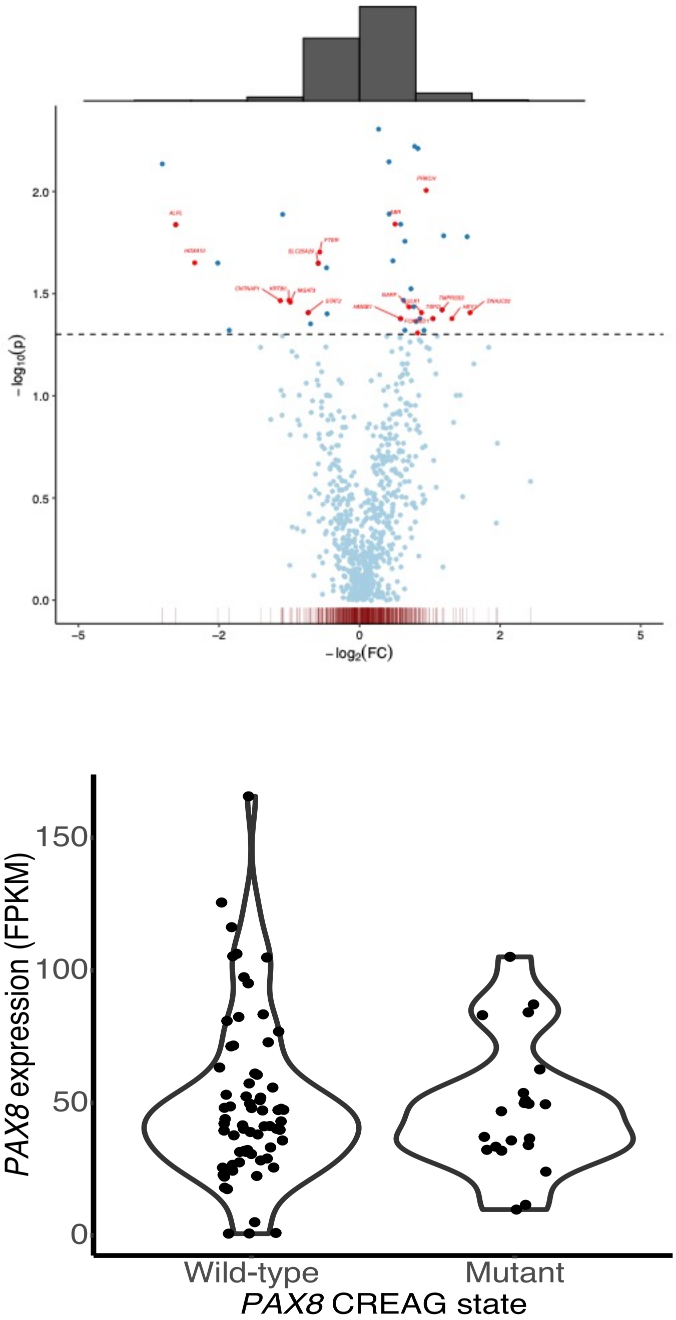


**Supplementary Figure 7. (a)** Volcano plot that shows the fold change of median gene expression (x-axis) and the significance value (y-axis) of the putative target gene of samples with overlapping single nucleotide variants in a CREAG vs. wild-type samples. PAX8 target genes are highlighted in red. **(b)** PAX8 expression for PAX8 CREAG mutant samples (n=21) and wild-type (n=68).

**Supplementary Table 1.** Samples included in H3K27ac ChIP-seq analysis

| Sample ID | Hosp | Tissue | Cancer site | Age | Race | Ethnicity | Grade | Stage |
|---|---|---|---|---|---|---|---|---|
| CCOC-1 | USC | Clear cell | Ovary | 45 | Asian | Non-Hispanic | 3 | IIIC |
| CCOC-2 | USC | Clear cell | Ovary | 47 | White | Hispanic or Latino | 3 | IIIC |
| CCOC-3 | CSMC | Clear cell | Ovary | 61 | | | 3 | IIIC |
| CCOC-4 | CSMC | Clear cell | Ovary | 38 | | | 3 | IIIB |
| CCOC-5 | CSMC | Clear cell | Ovary | 52 | | | 3 | IIIB |
| EnOC-1 | USC | Endometrioid | Ovary | 64 | Unknown | Hispanic or Latino | 3 | IV |
| EnOC-2 | USC | Endometrioid | Ovary | 50 | White | Hispanic or Latino | 1 | IB |
| EnOC-3 | USC | Endometrioid | Ovary | 35 | White | Hispanic or Latino | 2 | IC |
| EnOC-4 | USC | Endometrioid | Ovary | 42 | White | Hispanic or Latino | 2 | IC |
| EnOC-5 | USC | Endometrioid | Ovary | 41 | White | Hispanic or Latino | 1 | IC |
| HGSOC-1 | USC | High-grade serous | Ovary | 49 | Asian | Non-Hispanic | 3 | IIIC |
| HGSOC-2 | USC | High-grade serous | Ovary | 52 | Unknown | Hispanic or Latino | 3 | IIA |
| HGSOC-3 | USC | High-grade serous | Ovary | 45 | Unknown | Hispanic or Latino | 3 | IC |
| HGSOC-4 | USC | High-grade serous | Ovary | 56 | White | Hispanic or Latino | 3 | IV |
| HGSOC-5 | USC | High-grade serous | Ovary | 43 | White | Hispanic or Latino | 3 | IIIC |
| MOC-1 | USC | Mucinous | Ovary | 38 | White | Hispanic or Latino | 1 | IC |
| MOC-2 | USC | Mucinous | Ovary | 27 | Asian | Non-Hispanic | 1 | IA |
| MOC-3 | USC | Mucinous | Ovary | 47 | Black or African American | Non-Hispanic | 1 | IA |
| MOC-4 | USC | Mucinous | Ovary | 55 | Black or African American | Non-Hispanic | 1 | IC |
| MOC-5 | CSMC | Mucinous | Ovary | 76 | | | 1 | IA |

**Status key**
NED=no evidence of disease
AWD=alive w disease
DOD=Died of Disease

**PAX8 key**
S=strong
M=moderate
W=weak
NEG=no staining

**Supplementary Table 2.** Number of super-enhancer (SEs) called for each ovarian cacner sample.

| Sample name | Number of super-enhancers |
|---|---|
| CCOC-1 | 1161 |
| CCOC-2 | 1171 |
| CCOC-3 | 992 |
| CCOC-4 | 1116 |
| CCOC-5 | 1945 |
| EnOC-1 | 1162 |
| EnOC-2 | 1874 |
| EnOC-3 | 653 |
| EnOC-4 | 1121 |
| EnOC-5 | 1186 |
| HGSOC-1 | 1423 |
| HGSOC-2 | 1361 |
| HGSOC-3 | 1250 |
| HGSOC-4 | 841 |
| HGSOC-5 | 1511 |
| MOC-1 | 880 |
| MOC-2 | 1138 |
| MOC-3 | 1489 |
| MOC-4 | 1077 |
| MOC-5 | 1547 |

**Supplementary Table 3.** Frequently mutated regulatory elements across all histotypes.

| Name | seqnames | start | end | nj | nSNV | nSamples | p.value | FDR | Histotype |
|---|---|---|---|---|---|---|---|---|---|
| POLR3E promoter | chr16 | 22307782 | 22308738 | 957 | 9 | 8 | 2.4E-09 | 3.7E-05 | HGSOC |
| KLF6 promoter | chr10 | 3827366 | 3829505 | 2140 | 9 | 9 | 8.2E-08 | 6.2E-04 | HGSOC |
| CHCHD6 promoter | chr3 | 126422837 | 126423886 | 1050 | 6 | 6 | 2.3E-06 | 1.2E-02 | HGSOC |
| 17q25.3 | chr17 | 76641007 | 76642088 | 1082 | 4 | 4 | 9.2E-06 | 5.3E-02 | CCOC |
| DKC1 promoter | chrX | 153990093 | 153992603 | 2511 | 6 | 5 | 1.2E-05 | 5.3E-02 | CCOC+EnOC |
| C16orf87 promoter | chr16 | 46863901 | 46865831 | 1931 | 6 | 4 | 1.5E-05 | 7.9E-02 | EnOC |
| 6p22.1 | chr6 | 27869794 | 27871361 | 1568 | 7 | 6 | 2.2E-05 | 7.8E-02 | HGSOC |
| 12p12.2 | chr12 | 20119857 | 20120816 | 960 | 5 | 5 | 2.6E-05 | 7.8E-02 | HGSOC |
| AKT2 promoter | chr19 | 40747862 | 40749385 | 1524 | 3 | 3 | 2.9E-05 | 7.0E-02 | CCOC |
| Xq12 | chrX | 64917131 | 64918820 | 1690 | 6 | 6 | 3.3E-05 | 8.3E-02 | HGSOC |
| 10q21.2 | chr10 | 61623943 | 61624516 | 574 | 4 | 4 | 5.6E-05 | 1.2E-01 | HGSOC |
| CA5BP1 promoter | chrX | 15692029 | 15694894 | 2866 | 4 | 4 | 6.9E-05 | 1.5E-01 | EnOC |
| Xp11.23 | chrX | 47055131 | 47056180 | 1050 | 3 | 3 | 8.3E-05 | 1.5E-01 | EnOC |
| 1q42.2 | chr1 | 232764866 | 232766138 | 1273 | 5 | 5 | 9.6E-05 | 1.6E-01 | HGSOC |
| 2p21 | chr2 | 43198657 | 43199408 | 752 | 3 | 3 | 1.1E-04 | 1.6E-01 | CCOC |
| 17q25.2 | chr17 | 74998390 | 74999196 | 807 | 3 | 3 | 1.4E-04 | 1.7E-01 | EnOC |
| 20q13.2 | chr20 | 52684179 | 52685428 | 1250 | 3 | 3 | 1.4E-04 | 1.6E-01 | EnOC |
| SLIT3 promoter | chr5 | 168727171 | 168728476 | 1306 | 3 | 3 | 1.6E-04 | 1.6E-01 | EnOC |
| 20q13.33 | chr20 | 62442044 | 62442933 | 890 | 3 | 3 | 1.8E-04 | 2.0E-01 | CCOC |
| 2q35 | chr2 | 218844095 | 218845477 | 1383 | 3 | 3 | 1.9E-04 | 1.6E-01 | EnOC |
| 9q21.12 | chr9 | 73020654 | 73022163 | 1510 | 3 | 3 | 2.4E-04 | 1.8E-01 | EnOC |
| HUWE1 promoter | chrX | 53709258 | 53711891 | 2634 | 4 | 4 | 2.8E-04 | 2.2E-01 | CCOC |
| 1p31.3 | chr1 | 61522297 | 61523940 | 1644 | 3 | 3 | 3.1E-04 | 2.0E-01 | EnOC |
| 17q21.32 | chr17 | 46078755 | 46081470 | 2716 | 4 | 4 | 3.1E-04 | 2.2E-01 | EnOC |
