# Supplementary material for: Non-coding somatic mutations converge on the PAX8 pathway in ovarian cancer
Source: Nat Commun. 2020 Apr 24;11:2020. doi: 10.1038/s41467-020-15951-0 (PMC7181647; doi:10.1038/s41467-020-15951-0)
Supplement: Supplementary file 3 — Description of Additional Supplementary Files [file 41467_2020_15951_MOESM3_ESM.pdf]

Description of Additional Supplementary Files

File Name: Supplementary Data 1
Description: Gene sets associated with EOC histotype-specific and common regulatory elements.
