# Supplementary material for: Non-coding somatic mutations converge on the PAX8 pathway in ovarian cancer
Source: Nat Commun. 2020 Apr 24;11:2020. doi: 10.1038/s41467-020-15951-0 (PMC7181647; doi:10.1038/s41467-020-15951-0)
Supplement: Supplementary file 5 — Reporting Summary [file 41467_2020_15951_MOESM5_ESM.pdf]

nature research

Corresponding author(s): Kate Lawrenson, PhD

Last updated by author(s): Mar 24, 2020

Reporting Summary

Nature Research wishes to improve the reproducibility of the work that we publish. This form provides structure for consistency and transparency in reporting. For further information on Nature Research policies, see Authors & Referees and the Editorial Policy Checklist .

Statistics

For all statistical analyses, confirm that the following items are present in the figure legend, table legend, main text, or Methods section.

n/a	Confirmed	
[ ]	[x]	The exact sample size (*n*) for each experimental group/condition, given as a discrete number and unit of measurement
[ ]	[x]	A statement on whether measurements were taken from distinct samples or whether the same sample was measured repeatedly
[ ]	[x]	The statistical test(s) used AND whether they are one- or two-sided<br>*Only common tests should be described solely by name; describe more complex techniques in the Methods section.*
[x]	[ ]	A description of all covariates tested
[ ]	[x]	A description of any assumptions or corrections, such as tests of normality and adjustment for multiple comparisons
[ ]	[x]	A full description of the statistical parameters including central tendency (e.g. means) or other basic estimates (e.g. regression coefficient) AND variation (e.g. standard deviation) or associated estimates of uncertainty (e.g. confidence intervals)
[ ]	[x]	For null hypothesis testing, the test statistic (e.g. *F*, *t*, *r*) with confidence intervals, effect sizes, degrees of freedom and *P* value noted<br>*Give P values as exact values whenever suitable.*
[x]	[ ]	For Bayesian analysis, information on the choice of priors and Markov chain Monte Carlo settings
[x]	[ ]	For hierarchical and complex designs, identification of the appropriate level for tests and full reporting of outcomes
[ ]	[x]	Estimates of effect sizes (e.g. Cohen's *d*, Pearson's *r*), indicating how they were calculated

Our web collection on statistics for biologists contains articles on many of the points above.

Software and code

Policy information about availability of computer code

Data collection	ChIP-seq processing (AQUAS 0.3.3, https://github.com/kundajelab/chipseq_pipeline); RNA-seq processing (STAR 2.5.16, Picard 1.138, featureCounts 1.5.0-p1).
Data analysis	R 3.6.0; R Bioconductor packages (ComplexHeatmap 2.0.0, DESeq2 1.24.0, DiffBind 2.12.0, GenomicRanges 1.36.1, TxDb.Hsapiens.UCSC.hg19.knownGene 3.2.2, VariantAnnotation 1.30.1, biomaRt 2.40.5, edgeR 3.26.8, org.Hs.eg.db 3.8.2, rtracklayer 1.44.4).<br><br>Custom code is available at the Lawrenson Lab GitHub repository [https://github.com/lawrenson-lab/Non-coding-Somatic-Mutations-in-OvCa]

For manuscripts utilizing custom algorithms or software that are central to the research but not yet described in published literature, software must be made available to editors/reviewers. We strongly encourage code deposition in a community repository (e.g. GitHub). See the Nature Research guidelines for submitting code & software for further information.

Data

Policy information about availability of data

All manuscripts must include a data availability statement. This statement should provide the following information, where applicable:

- Accession codes, unique identifiers, or web links for publicly available datasets
- A list of figures that have associated raw data
- A description of any restrictions on data availability

The RNA-sequencing and ChIP sequencing data have been deposited in the GEO database under the accession code GSE121103 [https://www.ncbi.nlm.nih.gov/geo/query/acc.cgi?acc=GSE121103]. The whole-genome sequencing data is available from the PCAWG database under the search “Primary Site=Ovary” and “Software=PCAWG SNV-MNV callers” [https://dcc.icgc.org/pcawg]. All the other data supporting the findings of this study are available within the article and its supplementary information files and from the corresponding author upon reasonable request. A reporting summary for this article is available as a Supplementary

Information file.

Field-specific reporting

Please select the one below that is the best fit for your research. If you are not sure, read the appropriate sections before making your selection.

☒ Life sciences ☐ Behavioural & social sciences ☐ Ecological, evolutionary & environmental sciences

For a reference copy of the document with all sections, see nature.com/documents/nr-reporting-summary-flat.pdf

Life sciences study design

All studies must disclose on these points even when the disclosure is negative.

Sample size	The tissue specimens were either collected as part of the USC Jean Richardson Gynecologic Tissue and Fluid Repository or as part of the Women's Cancer Biobank at Cedars-Sinai Medical Center, from women with ovarian cancer diagnosis. 20 ovarian cancer samples were profiled using H3K27ac ChIP-seq, and we observed saturation of new enhancers at ~16 samples, providing evidence that we have covered most of the active chromatin regions with these samples. 232 WGS samples were collected from PCAWG or UBC, which is the largest OC WGS dataset available, providing the largest statistical power for data analysis.
Data exclusions	One Endometrioid OC WGS sample was excluded since it presented an outstanding number of mutations. One Endometrioid OC RNA-seq sample was not included due to technical difficulties to perform the data collection. The exclusion of the WGS sample was established in the early phases of data analysis (when checking for mutation rate per sample), and the one EnOC sample was excluded for RNA-seq during the data generation phase.
Replication	PPP1R3B KD experiments were done in triplicates in two Clear Cell OC models, with good reproducibility. CRISPR-Cas9 mediated enhancer KO was performed in two High-Grade Serous OC cell lines, and single-cell derived clones were selected to measure the enhancer KO effect. All replicates/experiments are reported in the manuscript.
Randomization	Samples/patients were allocated in different OC histological subtypes based on pathology reports. Besides histological subtype, we did not control for other covariates, since histological subtype is the most important variable to our study.
Blinding	Blinding was not possible to this study, since we were trying to performed supervised analysis based on OC histological subtype, i.e., retrieve subtype-specific or frequently mutated regulatory elements.

Reporting for specific materials, systems and methods

We require information from authors about some types of materials, experimental systems and methods used in many studies. Here, indicate whether each material, system or method listed is relevant to your study. If you are not sure if a list item applies to your research, read the appropriate section before selecting a response.

Materials & experimental systems

n/a	Involved in the study	
☐	☒	Antibodies
☐	☒	Eukaryotic cell lines
☒	☐	Palaeontology
☒	☐	Animals and other organisms
☐	☒	Human research participants
☒	☐	Clinical data

Methods

n/a	Involved in the study	
☐	☒	ChIP-seq
☒	☐	Flow cytometry
☒	☐	MRI-based neuroimaging

Antibodies

Antibodies used	H3K27ac antibody (DiAGenode, C15410196, Denville, NJ)
Validation	Validation provided by the manufacturer [https://www.diagenode.com/files/products/antibodies/Datasheet_H3K27ac_C15410196.pdf]

Eukaryotic cell lines

Policy information about cell lines

Cell line source(s)	JHOC-5 (RRID:CVCL_4640), RMG-II (RRID:CVCL_2803), SHIN-3 (RRID:CVCL_A670), UWB1.289 (RRID:CVCL_B079) and HEK293T (RRID:CVCL_0063).

Authentication	None of the cell lines used were authenticated.
Mycoplasma contamination	All cell lines tested negative for Mycoplasma contamination.
Commonly misidentified lines (See ICLAC register)	No commonly misidentified cell lines were used in the study.

Human research participants

Policy information about studies involving human research participants

Population characteristics	The main population characteristic is current diagnosis of Ovarian Cancer (Clear Cell, Endometrioid, High-Grade Serous or Mucinous), other variables are described in the Supplementary Tables.
Recruitment	The tissue specimens were either collected as part of the USC Jean Richardson Gynecologic Tissue and Fluid Repository or as part of the Women's Cancer Biobank at Cedars-Sinai Medical Center.
Ethics oversight	All tissues used were collected with informed consent and the approval of the institutional review boards of the University of Southern California and Cedars-Sinai Medical Center.

Note that full information on the approval of the study protocol must also be provided in the manuscript.

ChIP-seq

Data deposition

- [x] Confirm that both raw and final processed data have been deposited in a public database such as GEO.
- [x] Confirm that you have deposited or provided access to graph files (e.g. BED files) for the called peaks.

Data access links<br>*May remain private before publication.*	GEO link: https://www.ncbi.nlm.nih.gov/geo/query/acc.cgi?acc=GSE121103
Files in database submission	ChIP-seq: peak calls (narrowPeak) and FASTQ files<br>RNA-seq: FASTQ files and gene count matrix
Genome browser session (e.g. UCSC)	No longer applicable

Methodology

Replicates	We did not generated replicates for primary tissue ChIP-seq
Sequencing depth	Tissue ChIP-seq was 75 bp single-end sequencing, with an average 33.7 million mapped reads.
Antibodies	H3K27ac antibody (DiAGenode, C15410196, Denville, NJ)
Peak calling parameters	Default parameters for AQUAS pipeline, for Histone ChIP-seq
Data quality	We checked the number of uniquely mapped reads, cross-correlation metrics, and fraction of reads in peaks.
Software	AQUAS pipeline for ChIP-seq processing (https://github.com/kundajelab/chipseq_pipeline)
